# Genome-wide analysis of transposable elements and satellite DNA in *Humulus scandens*, a dioecious plant with XX/XY_1_Y_2_ chromosomes

**DOI:** 10.3389/fpls.2023.1230250

**Published:** 2023-10-16

**Authors:** Guo-Jun Zhang, Ke-Li Jia, Jin Wang, Wu-Jun Gao, Shu-Fen Li

**Affiliations:** ^1^ School of Basic Medical Sciences, Xinxiang Medical University, Xinxiang, China; ^2^ College of Life Sciences, Henan Normal University, Xinxiang, China; ^3^ SanQuan Medical College, Xinxiang Medical University, Xinxiang, China

**Keywords:** *Humulus scandens*, repetitive sequences, satellites, sex chromosome evolution, transposable elements (TEs)

## Abstract

Transposable elements (TEs) and satellite DNAs, two major categories of repetitive sequences, are expected to accumulate in non-recombining genome regions, including sex-linked regions, and contribute to sex chromosome evolution. The dioecious plant, *Humulus scandens*, can be used for studying the evolution of the XX/XY_1_Y_2_ sex chromosomes. In this study, we thoroughly examined the repetitive components of male and female *H. scandens* using next-generation sequencing data followed by bioinformatics analysis and florescence *in situ* hybridization (FISH). The *H. scandens* genome has a high overall repetitive sequence composition, 68.30% in the female and 66.78% in the male genome, with abundant long terminal repeat (LTR) retrotransposons (RTs), including more Ty3/*Gypsy* than Ty1/*Copia* elements, particularly two Ty3/*Gypsy* lineages, Tekay and Retand. Most LTR-RT lineages were found dispersed across the chromosomes, though CRM and Athila elements were predominately found within the centromeres and the pericentromeric regions. The Athila elements also showed clearly higher FISH signal intensities in the Y_1_ and Y_2_ chromosomes than in the X or autosomes. Three novel satellite DNAs were specifically distributed in the centromeric and/or telomeric regions, with markedly different distributions on the X, Y_1_, and Y_2_ chromosomes. Combined with FISH using satellite DNAs to stain chromosomes during meiotic diakinesis, we determined the synapsis pattern and distinguish pseudoautosomal regions (PARs). The results indicate that the XY_1_Y_2_ sex chromosomes of *H. scandens* might have originated from a centric fission event. This study improves our understanding of the repetitive sequence organization of *H. scandens* genome and provides a basis for further analysis of their chromosome evolution process.

## Introduction

Plant genomes typically consist of a large number of various repetitive DNA sequences. For example, they represent approximately 85% and more than 90% of the maize and onion genomes, respectively (Schnable et al., 2009; Fu et al., 2019). According to their structural arrangement and sequence composition, repetitive DNAs can be divided into two major groups: tandem repeats and transposable elements (TEs) (reviewed in Biscotti et al., 2015). Tandem repeats are arrays of non-coding sequences arranged in tandem. According to the monomer length, tandem repeats are usually classified into microsatellites (2−7 bp), minisatellites (tens of bp), and satellites (hundreds of bp). The satellite DNAs predominately cluster at specific positions on the chromosomes, such as (peri)centromeres, (sub)telomeres, and other heterochromatic regions, making them ideal markers for cytogenetic analysis (He et al., 2015; Kirov et al., 2017; Lang et al., 2019; Li et al., 2019). TEs are elements that have the unique ability to mobilize from one position of a genome to another. Based on whether using RNA as a transposition intermediate, TEs are divided into two major classes: retrotransposons transposing via copy-and-paste mechanism and DNA transposons using cut-and-paste transposition mode. Due to the transposition mechanism, retrotransposons are the most prevalent TEs in plant genomes. Of these, the long terminal repeat (LTR) retrotransposons, primarily Ty1/*Copia* and Ty3/*Gypsy*, were shown to be the most frequent in the plant genomes (Neumann et al., 2019).

According to popular belief, it is impossible to understand how eukaryotes’ complex genomes are shaped by evolutionary mechanisms without extensive examination of repetitive genomic sequences (reviewed in Shapiro and von Sternberg, 2005; Slotkin and Martienssen, 2007). Angiosperm plants are particularly prone to this due to the large proportion of repetitive sequences in the genome and the significant contribution of them to the extraordinary variation in genome sizes among different taxa (Piegu et al., 2006; Pellicer et al., 2021; Sader et al., 2021). Even though most repetitive elements tend to be selfish, growing evidence presents that repetitive sequences play a variety of roles, such as influencing the organization and stability of the genome (Bennetzen and Wang, 2014), mediating chromosomal rearrangement (reviewed in George and Alani, 2012), chromatin modulation (reviewed in Ohtani and Iwasaki, 2021), modification of gene expression (Wyler et al., 2020), and shaping phenotypic variation (Cai et al., 2022).

The majority of flowering plants are hermaphrodites, e.g., individual plants contain bisexual flowers harboring both pistil and stamen, and with just around 6% being dioecious, that is, plants with unisexual flowers on different individuals (reviewed in Renner and Ricklefs, 1995). These dioecious plants have multiple sex-determining mechanisms, including the XY and the ZW sex chromosome systems, as well as the sex index, i.e., the ratio of X chromosomes to autosome sets (X:A) (Baránková et al., 2020). In either case, the sex chromosomes are thought to be evolved from a pair of autosomes. Several events occurred during the sex chromosome evolutionary process, such as the formation of sex-determining gene(s), recombination inhibition, TE amplification, and Y chromosome degeneration (reviewed in Charlesworth, 2015). Among them, the accumulation of repetitive sequences is a dominant feature of the non-recombining region of the sex chromosome. This phenomenon has been observed in a variety of dioecious plants, for instance in *Carica papaya* (Wang et al., 2012; VanBuren and Ming, 2013), *Rumex acetosa* (Jesionek et al., 2021), and *Spinacia oleracea* (Li et al., 2019). These repetitive sequences have been postulated to be important driving forces for the evolution of sex chromosomes (Yang et al., 2021).

*Humulus scandens* is a climbing dioecious herbaceous plant belonging to the Cannabaceae family. The chromosome numbers of males and females are different; females have 2n = 16 = 14 + XX, whereas males have 2n = 17 = 14 + XY_1_Y_2_. The gender of *H. scandens* is governed by the X:A ratio; with an X:A ratio of 1.0 for females, and a ratio of 0.5 for males (Shephard et al., 2000). The multiple sex chromosome system (XX/XY_1_Y_2_) and the X:A ratio sex determination system make *H. scandens* an ideal species for examining sex chromosome evolution and sex determination mechanism. The two Y chromosomes are approximately the same size as X chromosome (Grabowska-Joachimiak et al., 2006; Alexandrov et al., 2012). GISH painting of the Y chromosomes showed that the male-specific region of the Y chromosome (MSY) spans the bulk of the Y chromosomes, suggesting advanced phases of sex chromosome evolution in *H. scandens*, as well as its relatives in the same family, *H. lupulus* and *Cannabis sativa* (Razumova et al., 2023). To date, limited studies have reported the repetitive elements of *H. scandens* (Alexandrov et al., 2012). The abundance and distribution of repetitive sequences, as well as role of repetitive sequences in the sex chromosome evolution of *H. scandens* has not been thoroughly examined. To gain a better understanding of the genome structure of *H. scandens*, we extensively analyzed the repetitive components of male and female *H. scandens* by combining next-generation sequencing, bioinformatics analysis, and florescence *in situ* hybridization (FISH). We first used a graph-based clustering strategy to identify and annotate repetitive sequences based on next-generation sequencing data. Then we examined the distribution patterns of different groups of TEs and satellite DNAs using FISH analysis. Our research offers a crucial foundation for comprehending the repeat elements in *H. scandens*.

## Materials and methods

### Plant material and DNA extraction

Plants of *H. scandens* were cultivated in a garden field at Henan Normal University. The genders were determined by observation of the morphology of flowers. Whole genomic DNA was extracted from three male and three female individuals using the cetyl trimethylammonium bromide method (Doyle and Doyle, 1987).

### DNA library preparation, high-throughput sequencing and repetitive DNA identification

At least 3 μg genomic DNA of each sample was fragmented, end repaired, phosphorylated, and ligated with adapters. Then 300−400 bp fragments were selected and PCR amplified to construct paired-end library. The libraries were sequenced on Illumina NovaSeq 6000 platform in paired-end, 150-bp mode. The raw reads (DRR024400, DRR024402, DRR024404, DRR024405, DRR024456, and DRR024457) of *H. lupulus*, a close relative of *H. scandens*, were downloaded from the NCBI SRA database. For the preprocessing of raw sequence data, the FASTQ files were quality-controlled, filtered using HTQC with default parameters (v1.92.1) (Yang et al., 2013), then a custom perl script was used to filter out the reads with N and transform into FASTA format. Then, for each sample, a randomly chosen dataset comprising 2,000,000 paired-end reads, which represented approximately 0.34× of the genome, was used for further analysis. We clustered, assembled, and annotated all selected reads with the help of RepeatExplorer platform (http://www.repeatexplorer.org, Novák et al., 2020) using the Green Plants (Viridiplantae) database (Neumann et al., 2019). To categorize LTR retrotransposons into different lineages, their RT sequences were analyzed using DANTE within the RepeatExplorer platform. After eliminating duplicated sequences using CD-hit (Li and Godzik, 2006), these RT sequences were aligned with MUSCLE (Edgar, 2004), and phylogenetic trees were generated using FastTree (Price et al., 2010). FigTree software was used to draw and modify the trees.

### Satellite DNA identification

The TAREAN tool embedded in RepeatExplorer was adopted to detect satellite DNA using the same samples described above (Novák et al., 2017). The high-confidence satellites were identified. The logo for the satellites was drawn by Web-Logo (Crooks et al., 2004).

### FISH probe design

For LTR retrotranposons, the RT domains of each lineage were amplified using specific primers (**Table S1**). The gel electrophoresis, cloning, and sequence validation were performed following a previous study (Li et al., 2019). Finally, clones with high sequence similarity to the respective contigs were amplified and labeled with Texas-red-dCTP (PerkinElmer, Waltham, Massachusetts, USA) utilizing the nick translation approach. For satellite DNA, the monomers were identified, and 50 bp of the monomer was randomly chosen and tagged directly with Texas Red-X (Invitrogen, Shanghai, China) in the synthesis process. 45S rDNA was also labeled with Chroma Tide Alexa Fluor 488-5-dUTP (Invitrogen) to aid in chromosome identification.

### Mitotic and meiotic chromosome spreads preparation

For mitotic chromosome spread preparation, the branches of both male and female plants were cut and placed in water until the new roots were grown. When the roots were grown to 1–1.5 cm, they were cut and treated in nitrous oxide at 10.9 atm pressure, fixed in a 90% acetic acid solution for 10 min. For meiotic chromosome spread preparation, male inflorescences were fixed in Carnoy’s fixative and stored in 70% ethanol. The immature flowers with a diameter of 1.3–1.5 mm were selected, and the anthers were picked out for further analysis. The enzyme digestion and drop spreading were completed according to earlier instructions (Li et al., 2019).

### FISH assays using LTR-RT domain and satellite probes

FISH analysis was carried out as previously described (Li et al., 2019). First, the slides with well-spread chromosomes or desired meiotic stages were UV cross-linked. Then the hybridization mixture (2×SSC, 1×TE, 200 ng labeled probe) was added to the slides, followed by denaturation in boiled water for 5 min. After that, the denatured chromosome slides with probes were incubated overnight at 37°C. Next, the slides were washed in 2×SSC for 5 min, and counterstained with DAPI solution. FISH signals were detected under an Olympus BX 63 fluorescence microscope with an ANDOR CCD.

### Statistical analysis

Pairwise comparisons were used to assess how the data groups differed from one another using Excel software. Least significance difference (LSD) analysis was performed on the data with a significance threshold of 0.05.

## Results

### Repeat proportion in *H. scandens* genome

**Figure 1:**
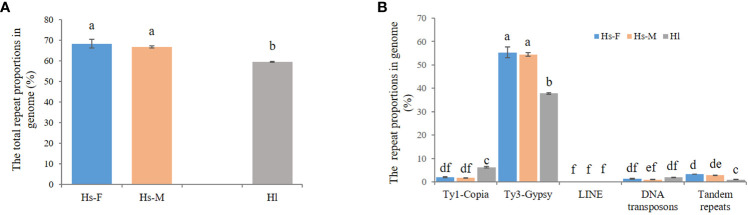
Comparison of repetitive sequences between the *H*. *scandens* and *H*. *lupulus* genomes. **(A)** The total repeat proportions in the *H*. *scandens* and *H*. *lupulus* genomes. Hs-F, female genome of *H*. *scandens*; Hs-M, male genome of *H*. *scandens*; Hl, *H*. *lupulus* genome. **(B)** The repeat proportion in the *H*. *scandens* and *H*. *lupulus* genomes. “a, b, c, d, e, f” means significance level by multiple comparison, *p*< 0.05.

Next-generation sequencing produced a total of approximately 20 Gb of raw sequencing data for three male and three female *H. scandens*. For each individual, a subset of 2,000,000 clean paired reads, representing about 34% of the genome, was chosen for repetitive sequence analysis. We also used sequencing reads of the *H. lupulus* genome for comparison analysis. **Table 1** shows the comparative proportions of different repeat types in the three genomes. The findings revealed a high composition of repetitive sequences in the *H. scandens* genome, with 68.30% in the female genome and 66.78% in the male genome. These values were significantly higher than those of the *H. lupulus* genome (59.45%) (**Figure 1A**). Similar to most other plant species, LTR-retrotransposons were the most prevalent group of *H. scandens* repeats, accounting for nearly 60% of the genome (when male and female values were averaged), followed by tandem repeats (3.06%), DNA transposons (1.16%), and long interspersed nuclear elements (LINEs) (<0.01%). Within the LTR retrotransposon superfamily, the proportion of Ty3/*Gypsy* elements was more than 30-fold greater than Ty1/*Copia* elements (**Table 1**; **Figure 1B**). In addition, about 3.35% of organelle DNAs were identified in the *H. scandens* genome.

**Table 1:** Repeat proportions (%) estimated in the *Humulus scandens* and *Humulus lupulus* genomes.

Repeats		Lineage/class	Hs-female				Hs-male				Hl			
			Hs-F1	Hs-F2	Hs-F3	Ave.	Hs-M1	Hs-M2	Hs-M3	Ave.	Hl-1	Hl-2	Hl-3	Ave.
LTR retroelements	Ty1*/Copia*	Ale	0.01	0.01	0.01	0.01	0.00	0.00	0.00	0.00	0.05	0.04	0.03	0.04
		Angela	0.27	0.13	0.25	0.22	0.28	0.20	0.25	0.24	3.27	3.25	3.81	3.44
		Ikeros	0.11	0.08	0.10	0.10	0.05	0.08	0.06	0.06	0.22	0.14	0.23	0.20
		SIRE	1.56	1.38	1.47	1.47	1.24	1.31	1.16	1.24	0.58	0.61	0.65	0.61
		TAR	0.10	0.09	0.12	0.10	0.09	0.12	0.08	0.10	1.99	2.00	1.87	1.95
		Tork	0.08	0.02	0.05	0.05	0.01	0.03	0.03	0.02	0.00	0.00	0.00	0.00
		**Subtotal**	**2.12**	**1.71**	**1.99**	**1.94**	**1.66**	**1.73**	**1.58**	**1.66**	**6.11**	**6.04**	**6.59**	**6.25**
	Ty3*/Gypsy*	Athila	8.24	8.04	7.84	8.04	8.66	8.94	8.61	8.74	0.79	0.80	0.88	0.82
		Ogre/Tat	0.02	0.05	0.02	0.03	0.03	0.01	0.03	0.02	0.00	0.00	0.00	0.00
		Retand	21.98	25.11	22.24	23.11	19.11	19.42	20.35	19.63	14.84	15.76	14.97	15.19
		CRM	0.16	0.14	0.16	0.15	0.16	0.17	0.16	0.16	0.35	0.36	0.35	0.35
		Galadriel	0.07	0.10	0.09	0.06	0.09	0.10	0.11	0.10	0.18	0.19	0.19	0.19
		Tekay	23.01	25.20	23.61	23.94	25.26	26.12	25.96	25.78	21.39	21.10	21.19	21.23
		**Subtotal**	**53.47**	**58.62**	**53.94**	**55.34**	**53.31**	**54.75**	**55.21**	**54.42**	**37.55**	**38.21**	**37.58**	**37.78**
	Unclassified LTR RTs		2.38	2.95	2.55	2.63	3.14	3.10	3.13	3.12	3.79	1.93	3.90	3.21
Other	LINE		0.00	0.01	0.01	0.01	0.00	0.00	0.00	0.00	0.00	0.00	0.00	0.00
	DNA transposons	EnSpm_CACTA	0.45	0.40	0.38	0.41	0.39	0.42	0.37	0.39	1.83	1.32	1.60	1.58
		hAT	0.02	0.02	0.01	0.02	0.01	0.02	0.01	0.01	0.05	0.04	0.04	0.04
		MuDR_Mutator	1.22	0.89	0.73	0.95	0.35	0.63	0.57	0.52	0.22	0.27	0.20	0.23
		**Subtotal**	**1.69**	**1.31**	**1.21**	**1.40**	**0.75**	**1.06**	**0.95**	**0.92**	**2.10**	**1.63**	**1.84**	**1.86**
	Tandem repeats	rDNA	1.06	1.53	1.34	1.31	0.90	0.93	0.93	0.92	0.16	0.15	0.15	0.15
		Satellite	2.26	1.77	2.04	2.02	2.05	1.71	1.85	1.87	0.85	0.80	0.66	0.77
		**Subtotal**	**3.32**	**3.30**	**3.38**	**3.33**	**2.95**	**2.64**	**2.78**	**2.79**	**1.01**	**0.95**	**0.81**	**0.92**
Total annotated repeats			**62.99**	**67.90**	**63.00**	**64.63**	**61.80**	**63.27**	**63.65**	**62.91**	**50.56**	**48.76**	**50.72**	**50.01**
Unclassified repeat			3.78	3.37	3.86	3.67	4.29	4.11	3.20	3.87	9.12	10.28	8.92	9.44
**Total**			**66.77**	**71.26**	**66.86**	**68.30**	**66.10**	**67.38**	**66.85**	**66.78**	**59.68**	**59.04**	**59.64**	**59.45**

Hs-female and Hs-male represent the female and male genomes of *H. scandens*, respectively. Hl represents the genome of *H. lupulus*. The bold values indicate the total value of “Ty1-*Copia*”, “Ty3-*Gypsy*”, “DNA transposons”, “Tandem repeats”, “Annotated repetitive sequences”, and all the repetitive sequences identified, respectively. “Ave.” means “the average value”.

**Figure 2:**
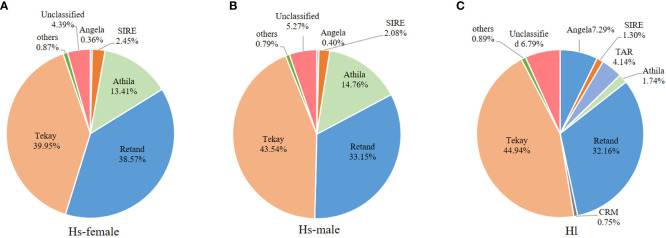
The proportions of different LTR-RT lineages in the *H*. *scandens* female **(A)** and male **(B)** genomes as well as the *H*. *lupulus* genome **(C)**.

For the comparison of different groups of repetitive sequences, the proportions of LTR-retrotransposons and tandem repeats of the *H. scandens* genome were all significantly higher than those of the *H. lupulus* genome (**Table 1**; **Figure 1B**). However, the DNA transposons showed a little higher proportions in the *H. lupulus* genome than in the *H. scandens* genome. Out of the LTR retrotransposons, the proportions of Ty3/*Gypsy* elements in the *H. scandens* genome were significantly higher than those in *H. lupulus*. By contrast, the *H. lupulus* genome showed higher proportions of Ty1/*Copia* elements than the *H. scandens* genome (**Table 1**; **Figure 1B**). Thus, the ratio of Ty3/*Gypsy* to Ty1/*Copia* in the *H. lupulus* genome was 6:1, compared with 30:1 in the *H. scandens* genome (**Table 1**). We also compared the proportions of various lineages of LTR retrotransposons (**Figure 2**). In the *H. scandens* genome, Tekay and Retand lineages belonging to the Ty3/*Gypsy* superfamily were prevalent, together accounting for nearly 80% of the LTR retrotransposable elements. The Athila lineage belonging to the Ty1/*Copia* superfamily ranked third (**Figures 2A, B**). In the *H. lupulus* genome, the Tekay and Retand lineages were also the more abundant elements, similar to the *H. scandens* genome; however, the third-ranked lineage was Angela (**Figure 2C**).

### Comparison of transposable elements between male and female *H. scandens* genomes

**Figure 3:**
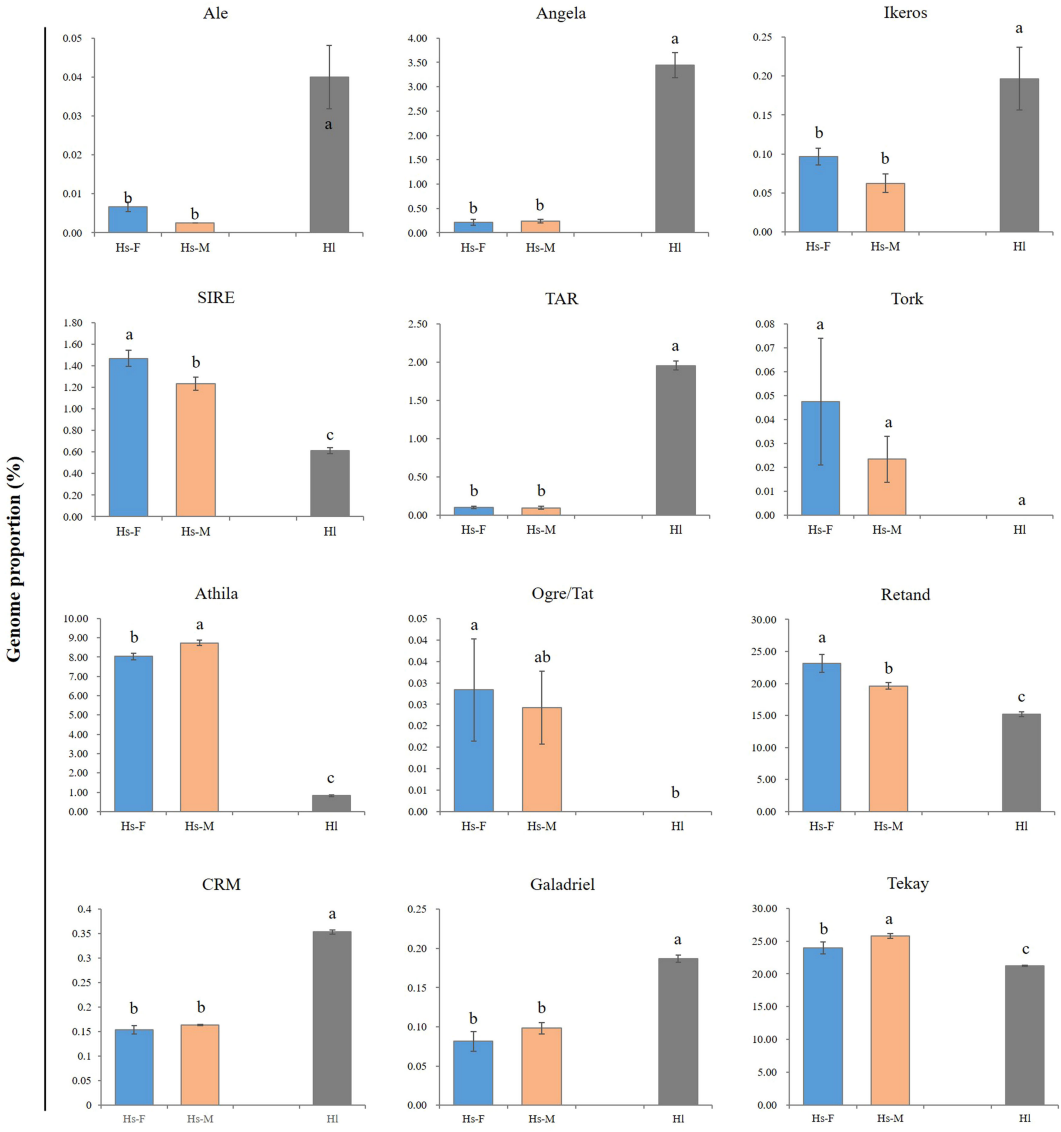
Comparison of the major LTR-RT lineages between male and female *H. scandens* as well as *H. lupulus* genomes. “a and b” mean significance level by multiple comparison, *p*< 0.05.

Generally, a conserved repeat proportion pattern was observed between the male and female *H. scandens* genomes. However, slight but significant differences were still observed in the proportions of several repeat groups between the two genomes with different genders, such as SIRE and Retand lineages, which all showed a higher proportion in female than in male genomes. By contrast, two other lineages, including Athila and Tekay, showed a higher proportion of males than females (**Figure 3**).

### Phylogenetic analysis of the lineages of LTR-RTs in *H. scandens* genome

**Figure 4:**
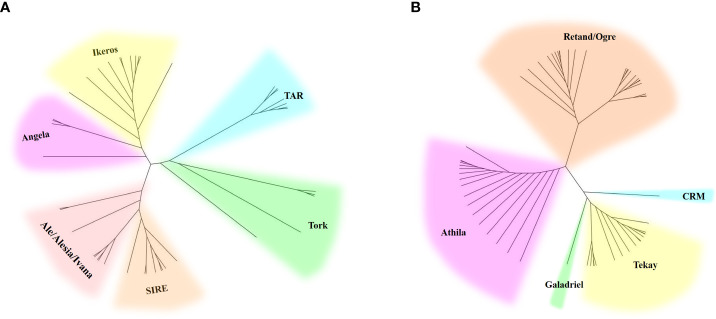
Phylogenetic analysis of Ty1/*Copia* **(A)** and Ty3/*Gypsy* **(B)** LTR-RT lineages represented by the LTR-RT domain protein sequences.

Two phylogenetic trees based on the RT sequences of the Ty1/*Copia* and Ty3/*Gypsy* LTR-RTs were created to ascertain their evolutionary relationships. As shown in the evolutionary dendrograms (**Figure 4**), the Ty1/*Copia* elements could be classified into three clades: one included elements belonging to Angela and Ikeros lineages, another consisted of TAR and Tork lineages, whereas SIRE, Ale, Alesia, and Ivana lineages grouped together to form the third clade. Among the Ty3/*Gypsy* elements, there were also three clades: Athila and Retand/Ogre each formed one clade, while the other three lineages, including CRM, Tekay, and Galadriel, formed the third clade. The Retand/Ogre lineage showed a high degree of variability and could be further segregated into two distinct groups.

### Chromosome distributions patterns of LTR-retrotransposons

**Figure 5:**
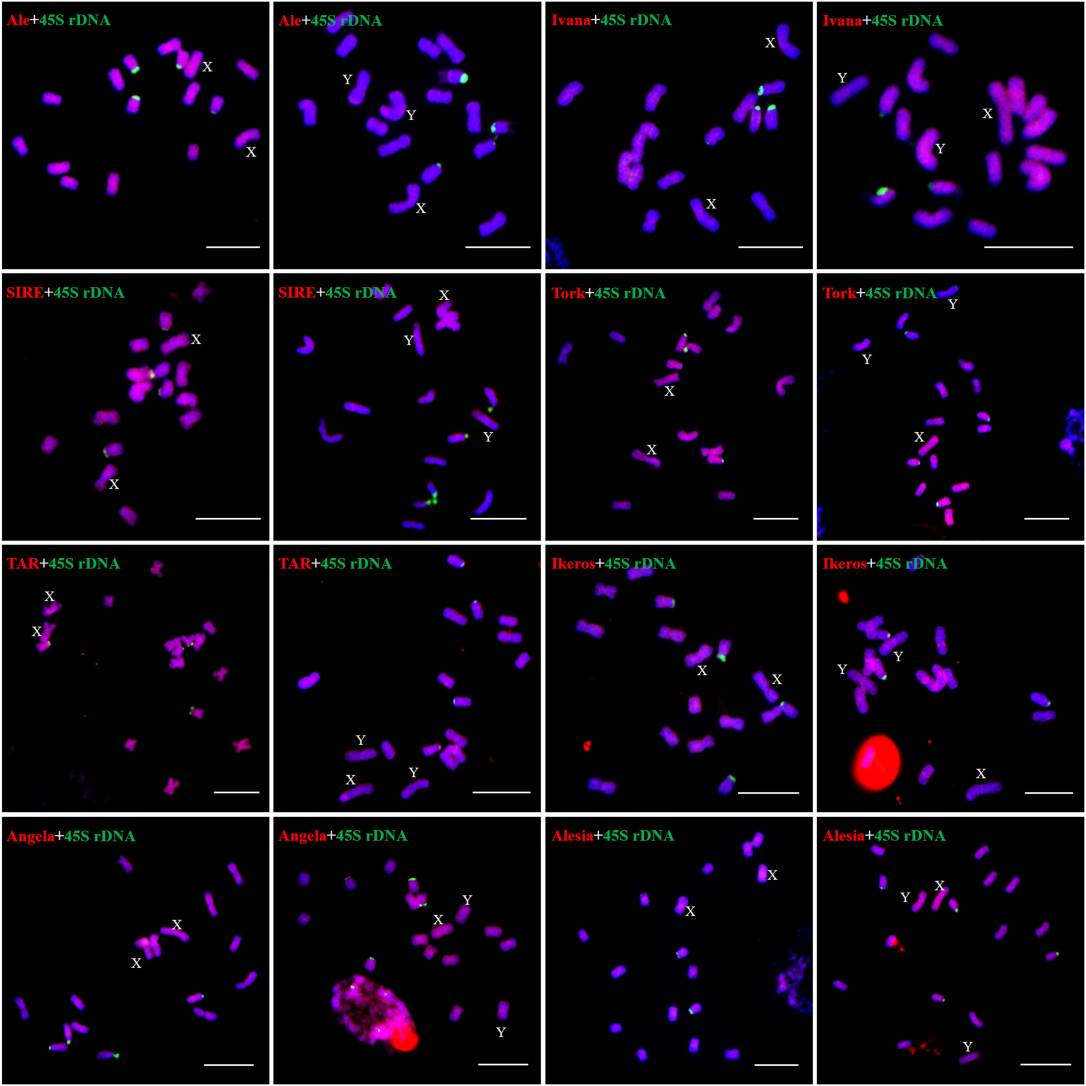
Distribution patterns of different LTR-RT lineages of Ty1/*Copia* elements on metaphase chromosomes of *H. scandens*. The lineage names and genders of individuals are presented inside each figure. The RT sequences of each lineage were labeled with Texas red (red), 45S rDNA was labeled with Chroma Tide Alexa Fluor 488 (green), and the chromosomes were counterstained with DAPI (blue). Arrows indicate the sex chromosomes. (Bars = 10 μm).

**Figure 6:**
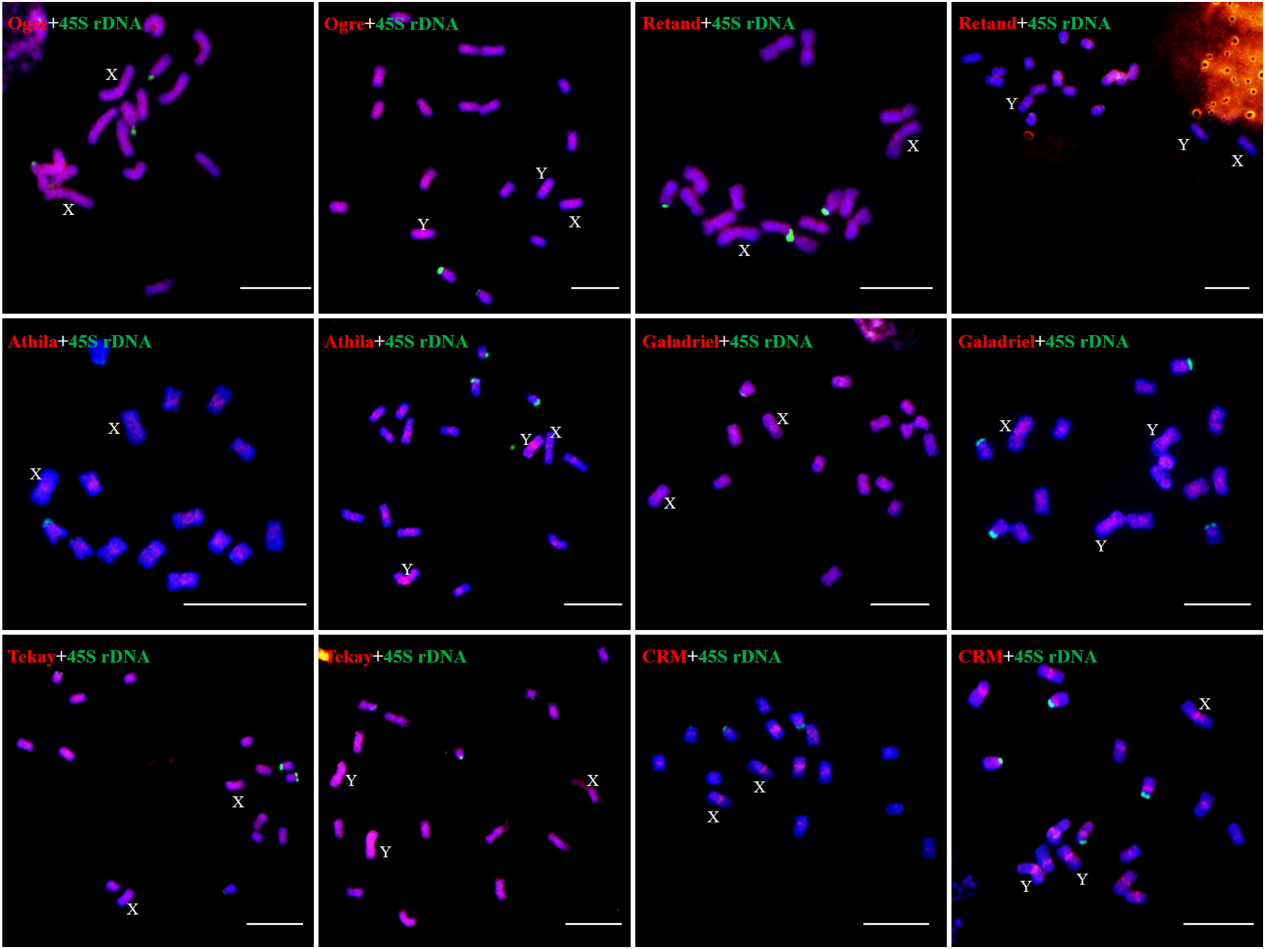
Distribution patterns of different LTR-RT lineages of Ty3/*Gypsy* elements on metaphase chromosomes of *H. scandens*. (Bars = 10 μm).

We examined the chromosomal distribution patterns of each lineage of LTR-RTs by using mitotic-FISH analysis. The findings demonstrated that different lineages has varied patterns of chromosomal distribution. Most of the lineage-based elements were dispersed over all of the chromosomes; these included all eight lineages of the *Copia* superfamily and four lineages of the *Gypsy* superfamily (Ogre, Retand, Galadriel, and Tekay) (**Figures 5**, **6**). The other two lineages (Athila and CRM) mainly occupied the pericentromeric regions of all chromosomes. In addition, the Athila elements showed clearly higher signal intensity in the Y_1_ and Y_2_ chromosomes than in the X and autosomes.

### Identification of satellites and analysis of chromosomal locations

By using TAREAN, three clusters (CL33, CL37, and CL111) were identified as highly reliable satellite DNAs. Each of them was given the designations Hssat1, Hssat2, and Hssat3, respectively. The three clusters were all characterized by star-like graph topologies (**Figure S1**). The monomer of Hssat1 was about 312 bp. A search of GenBank yielded no matches to other known sequences. The other two satellites, Hssat2 and Hssat3, demonstrated ~380 bp and ~122 bp, respectively. Like Hssat1, the clusters were all unknown or *H. scandens*-specific sequences.

**Figure 7:**
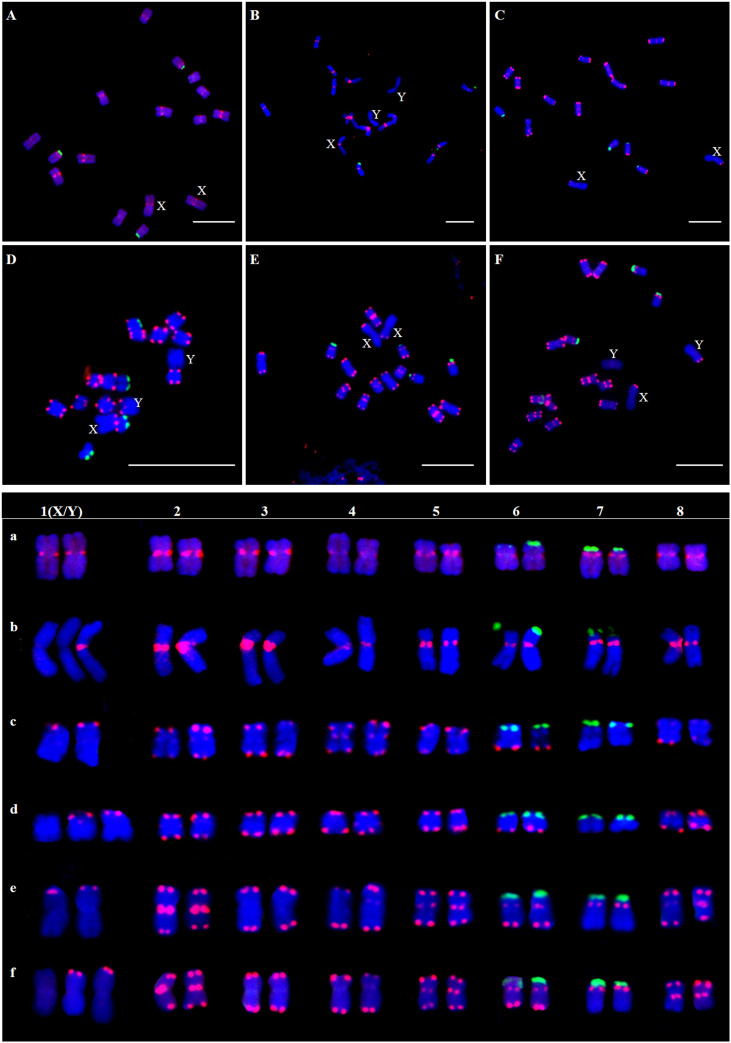
Localization of three satellites on metaphase chromosomes of female and male *H*. *scandens* using FISH analysis and karyotype of mitotic chromosomes based on FISH analysis of satellite DNAs in *H*. *scandens*. **(A)** Hssat1 on female chromosomes; **(B)** Hssat1 on male chromosomes; **(C)** Hssat2 on female chromosomes; **(D)** Hssat2 on male chromosomes; **(E)** Hssat3 on female chromosomes; **(F)** Hssat3 on male chromosomes. a-f show the karyograms of the metaphase chromosome spreads represented by the corresponding upper letters. (Bars = 10 μm).

**Figure 8:**
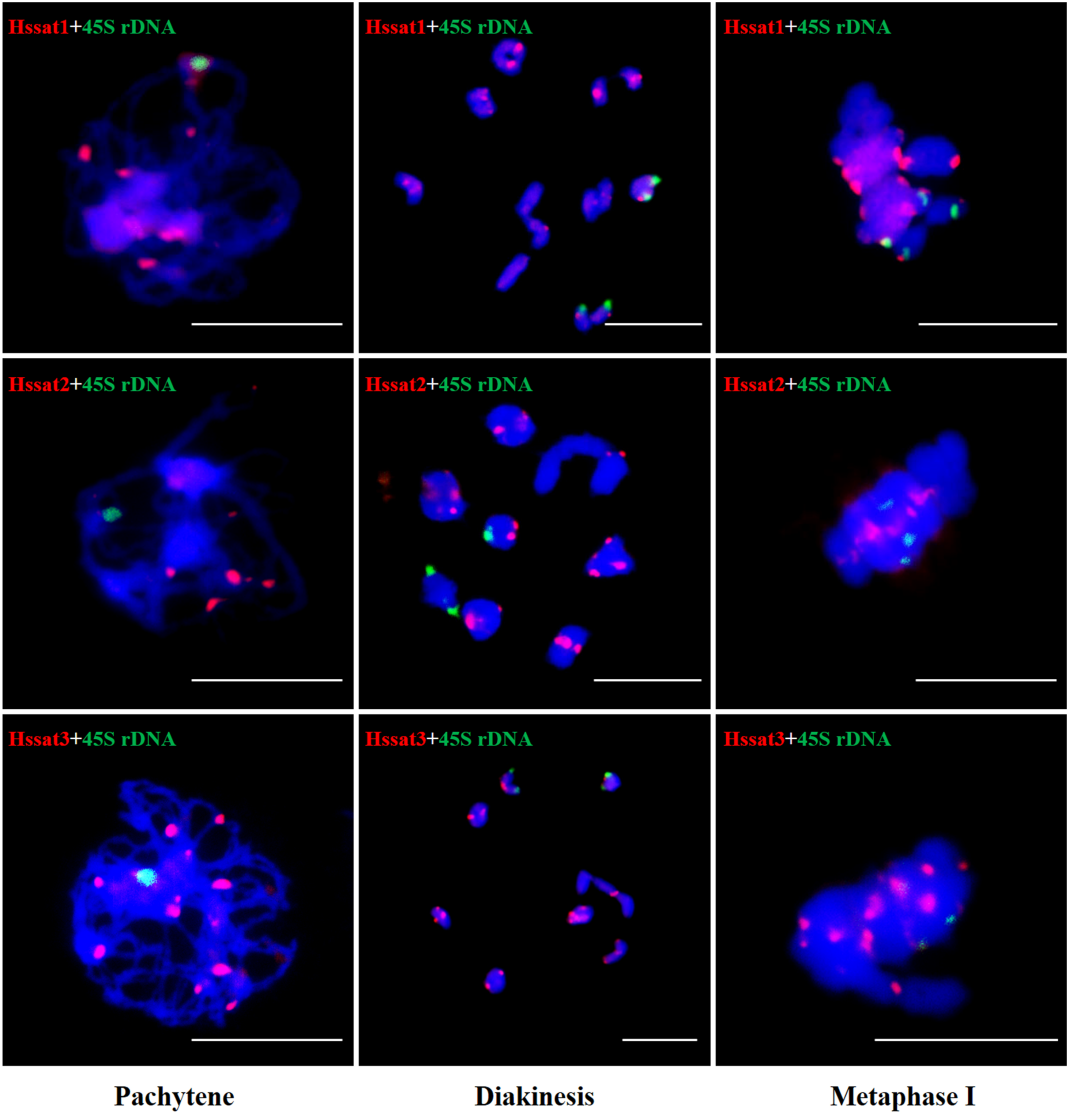
FISH analysis of three satellite DNAs on meiotic chromosomes in *H. scandens*. Three typical phases, including pachytene, diakinesis, and metaphase I are shown. (Bars = 10 μm).

The results showed that the signal of Hssat1 is distributed at the centromere of all chromosomes except for Y_1_ and Y_2_ chromosomes. Hssat2 was distributed specifically at the terminal position on metaphase chromosomes, but there is a large variation between male and female chromosomes. In the female plant, there were 4 pairs of chromosomes with signals at both ends, while in the male plant, there were 5 pairs. Even the chromosomes with signals at one end are inconsistent; for example, there were significant differences in the signals at the ends of chromosomes 6 and 7. In addition, it is worth noting that the signals of the two Y chromosomes were obviously inconsistent, one of which had a signal at one end while the other Y chromosome had no hybridization signal. The signal of Hssat3 showed more complex distribution characteristics, both at the end position and at the centromere position. In female *H. scandens*, the end positions of all chromosomes except one pair of autosomes could be seen, and obvious signals could be seen at the centromere positions of this pair of autosomes. There was no signal distribution on one Y chromosome of male *H. scandens*, and the signal of the other Y chromosome was also distributed at the end of one side, like the X chromosome (**Figures 7**, **8**).

## Discussion

### TE annotation of *H. scandens* genome

Repetitive sequences represent approximately 68% of the *H. scandens* genome, and this value is slightly but significantly higher than that of its close relative, the *H. lupulus* genome. Generally, the amount of repetitive sequences, particularly TEs, is positively correlated with the genome size of plants (Li et al., 2017). *H. scandens* (1.8 Gb) and *H. lupulus* (2.5 Gb) possess relatively large genomes, and the TE fraction proportions are generally in line with the trend. However, the genome of *H. scandens* is smaller than that of *H. lupulu*s, whereas the repetitive sequence fraction of *H. scandens* is higher than that of *H. lupulus*. This suggests that TEs were amplified more extensively in the *H. scandens* genome. Similar to other plant genomes, in the *H. scandens* genome, LTR-RTs were more abundant than DNA transposons, LINEs, and tandem repeats. The diversity and proliferation ability of LTR-RTs make them an important contributor to the structure, function, and evolution of the *H. scandens* genome. In particular, two Ty3/*Gypsy* lineages, Retand and Tekay, proliferated massively. This is similar to the phenomenon in many other plant genomes, in which one or several TE groups are significantly amplified. For example, differential lineage-specific proliferation of distinct families of transposable elements contributes greatly to genome size differences in *Gossypium* (Hawkins et al., 2006).

### Repetitive sequences and sex chromosome evolution of *H. scandens*

According to the general model of sex chromosome evolution, X and Y sex chromosomes are gradually differentiated from a pair of autosomes due to the suppression of recombination around the sex-determining locus. The accumulation of repetitive sequences can facilitate the recombination suppression of sex-determining loci and adjacent regions between X and Y chromosomes, eventually forming the sex-specific region. Furthermore, the formation of sex-specific regions can further recruit more repetitive sequences. Thus, repetitive sequence accumulation was a conspicuous feature of the sex chromosomes in both plants and animals (reviewed in Li et al., 2016). For instance, these findings were observed in papaya (VanBuren and Ming, 2013; VanBuren et al., 2015), *Rumex acetosa* (Mariotti et al., 2009), *Salix viminalis* (Almeida et al., 2020), *Salix dunnii* (He et al., 2021), so on and so forth. The TEs and TE-derived repetitive sequences are thought to be involved in almost all of the major evolutionary phases of sex chromosome evolution (reviewed in Li et al., 2016). Furthermore, TEs and associated repetitive sequences may influence plant sex determination and differentiation. For example, in *Populus deltoids*, a Ty3/*Gypsy* transposable element family member within the Y-linked region can generate long non-coding RNAs and act as a male promoter (Xue et al., 2020). In *H. scandens*, FISH analysis showed that the Athila elements accumulated more intensively in the Y_1_ and Y_2_ chromosomes. The Y chromosome-biased TEs may be involved in sex chromosome evolution in *H. scandens*.

However, the satellites did not show such an accumulation pattern. The signals of the three satellites are absent from one or two Y chromosomes. Such a phenomenon was also observed in other dioecious plant species. For instance, one family of the Ogre/Tat lineage is found on all autosomes and the X chromosome but not on the Y chromosome in *Silene latifolia* (Kubat et al., 2014). Due to this, it can be challenging to comprehend the way repetitive sequences and the evolution of plant sex chromosomes are related. According to the few reports that are currently available, the accumulation or depletion of some repetitive sequences appears to be species-specific, which is in keeping with the fact that sex chromosomes have evolved multiple times independently in different lineages of plants.

**Figure 9:**
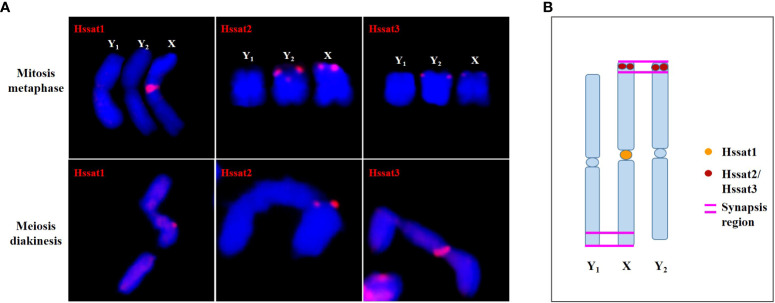
The synapsis pattern of the X, Y_1_, and Y_2_ sex chromosomes based on FISH signals of satellite DNAs. **(A)** The X, Y_1_, and Y_2_ sex chromosomes at mitosis metaphase and meiosis diakinesis stages with FISH signals of satellite DNAs. **(B)** Schematic diagram of synapsis pattern of the X, Y_1_, and Y_2_ chromosomes with FISH signals of satellite DNAs.

The satellite DNA probes showed typical signals located at the centromeric and/or telomeric regions. The X and Y chromosomes showed different signal distribution patterns, so the satellite DNA can be used as cytogenetic markers for identifying the X and Y chromosomes. Sex chromosome identification is crucial for cytological examinations and subsequent studies of sex chromosome evolution. Based on the cytogenetic markers, at the meiotic diakinesis stage, we observed obvious trivalents, showing Y_1_-X-Y_2_ connection mode. The synaptic region was the telomere position of two adjacent sex chromosomes connected end-to-end **Figure 9**. These results suggested that the sex-linked regions of *H. scandens* are large, which is in accordance with a recent study showing that the MSY covers the majority of the Y chromosomes (Razumova et al., 2023). These findings suggest advanced phases of sex chromosome evolution in *H. scandens.* In addition, the two Y chromosomes showed large differences because of the fact that the two Y chromosomes only pair at one telomeric end, and the majority of Y chromosomes are chromosome-specific regions. The FISH analysis using satellite probes also supports this perspective. Furthermore, combined with the fact that Hssat1 was specifically distributed on the centromeric regions of all the chromosomes except for the two Y chromosomes, we speculated that the XX-XY_1_Y_2_ sex chromosomes of *H. scandens* might have originated from a centric fission event. The centromere-specific satellite DNA might be lost during the centric fission event. However, we still need more evidence to support this speculation.

## Conclusions

In conclusion, this work allowed us to have a comprehensive view of the repetitive fraction of the nuclear genome of *H. scandens*, which is an important dioecious plant with XX/XY_1_Y_2_ chromosomes. We annotated the repetitive portion of both the male and female *H. scandens* genomes based on the RepeatExplorer platform and extensively compared the different groups of repetitive sequences among the male and female genomes of *H. scandens*, as well as a close relative, *H. lupulus*. We also analyzed the distribution patterns of major LTR-RT lineages and three satellite DNAs using FISH analysis. Based on the FISH results of satellite DNAs, we were able to determine the orientation position of the PARs, and the results also indicated that the XX-XY_1_Y_2_ sex chromosomes of *H. scandens* might have originated from a centric fission event. Our findings shed light on the genome structure and evolution of *H. scandens* and laid a foundation for future research into the sex chromosome evolution of *H. scandens*.

## Data availability statement

The datasets presented in this study can be found in online repositories. The names of the repository/repositories and accession number(s) can be found below: NCBI SRA database. BioProject accession number is PRJNA978042.

## Author contributions

S-FL and W-JG designed the experiments. G-JZ, K-LJ and JW conducted the study and processed the data. G-JZ wrote the manuscript. All authors discussed the results and revised the manuscript. All authors have read and approved the final manuscript. All authors contributed to the article and approved the submitted version.
