# Supplementary material for: Genome-wide analysis of transposable elements and satellite DNA in Humulus scandens, a dioecious plant with XX/XY1Y2 chromosomes
Source: Front Plant Sci. 2023 Oct 16;14:1230250. doi: 10.3389/fpls.2023.1230250 (PMC10614002; doi:10.3389/fpls.2023.1230250)
Supplement: Supplementary file 1 [file DataSheet_1.docx]

**TABLE S1** The primers used for amplification of the RT sequences of lineages in *H. scandens*

| Lineages | Primer sequence (5′-3′) |
| --- | --- |
| Ale | F: CAAAAAGTAGCGAAGAGGACC |
|  | R: AGCAGTTATTGACCCTCTTTG |
| Ivana | F: AGGAGACTAACTGCGTACATT |
|  | R: GCGAACAAGACCACTATCAGA |
| SIRE | F: GTCACGAGGAGAAGGTATGTA |
|  | R: CTCCCCCTTCATCTTTTGTTA |
| Tork | F: GCTAGAAGGGAAATCAAGGC |
|  | R: GCAATATCAGGTCTCGTACT |
| TAR | F: GTTCGAATTCCTGATCCTAAG |
|  | R: CTGCATAATAATCCCACGACA |
| Ikeros | F: CATAAGACGCATCACCCATA |
|  | R: GAGCCGAAAATGATCCAGAA |
| Angela | F: GGCTGACACATAGGGATACTT |
|  | R: GCAGAGAGAAGGTGTGGACTA |
| Alesia | F: AGTCCTTCCCTTCATGAGCAC |
|  | R: TGGGCTAGATTATGACGAGAC |
| Ogre | F: GTCTTCCTTGTAGTCCACGC |
|  | R: CACTGAGTTATTGGTAGACG |
| Retand | F: GGAGGACATGTATTGACTAT |
|  | R: TCTGTATGTTTCCTCGGCAA |
| Athila | F: AACCAACAAGAGAAGCCCAG |
|  | R: TGATGAAGCCTACCAACTCG |
| Galadriel | F: TGGCCGAGAAACTTGATCCT |
|  | R: ATGCCAGATCAACTCCCCAA |
| Tekay | F: GTGCATTGATCATCGAGAGT |
|  | R: CTTAACCTACGCCACATTCC |
| CRM | F: CCAAACCGACCAGCCTACAGA |
|  | R: TGTAAGTGGAGCGGCAATTGT |


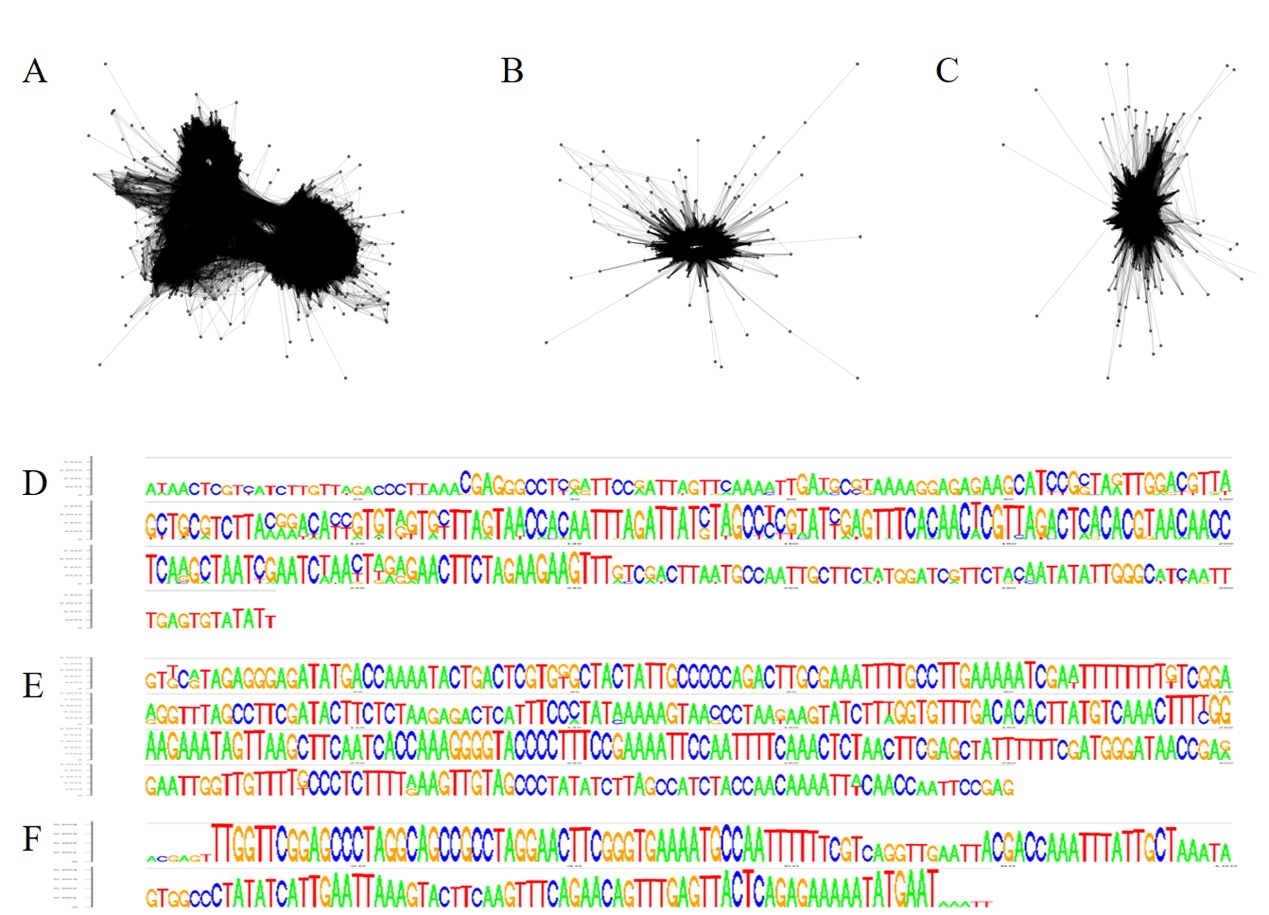


**FIGURE S1** Topological layout and consensus sequence of the three satellite DNAs. A, B, and C indicate the topology layouts of Hssat1, Hssat2, and Hssat3, respectively. D, E, and F show consensus sequences of Hssat1, Hssat2, and Hssat3, respectively.
